# Sudden onset of artery dissection in a 32-year-old woman with vascular Ehlers-Danlos syndrome due to psychological stress of her mother’s death: a case series

**DOI:** 10.1186/s40981-017-0094-5

**Published:** 2017-05-08

**Authors:** Yuichiro Shimoyama, Osamu Umegaki, Tomoyuki Agui, Noriko Kadono, Toshiaki Minami

**Affiliations:** 1 Department of Anesthesiology, Osaka Medical College, 2-7 Daigaku-machi, Takatsuki, Osaka 569-8686 Japan; 2 Department of Surgery, Osaka Medical College, Takatsuki, Japan

**Keywords:** Vascular Ehlers-Danlos syndrome, Vascular complication, Stent graft repair, Psychological care

## Abstract

**Background:**

Patients with vascular Ehlers-Danlos syndrome (EDS) are susceptible to significant vascular complications, such as aortic and visceral arterial ruptures, aneurysms, and dissection. We describe a case of repeated bleeding in a 57-year-old woman and a case of sudden onset of artery dissection in her daughter, both of whom were previously diagnosed with vascular EDS and managed at our institution.

**Case presentation:**

A 57-year-old woman was admitted to our emergency department due to sudden onset of left low back pain. Her past history included vascular EDS. An urgent abdominal computed tomography (CT) scan revealed a left-sided retroperitoneal hematoma and left external iliac artery dissection. Stent graft repair was performed. Five hours postoperatively, cardiac arrest occurred and resuscitation attempts failed. The 32-year-old daughter with genetically diagnosed vascular EDS was notified of the death of her mother during the customary end-of-life conference. Six hours after her mother’s death, she was admitted to our emergency department due to sudden onset of left low back pain. On examination, she was not in hypovolemic shock, and weak pulses were palpable in the bilateral dorsalis pedis. An urgent abdominal CT scan revealed a right-sided retroperitoneal hematoma around the right external iliac artery and left external iliac artery dissection. She was admitted to the intensive care unit and underwent conservative therapy consisting of bed rest and antihypertensive therapy with nicardipine. She developed no further vascular complications requiring surgical intervention and was discharged on the 21st hospital day.

**Conclusions:**

Vascular rupture can be fatal in patients with vascular EDS. This report underscores the importance of strategic management of vascular complications to prevent rupture, and the importance of psychological care for the bereaved family given the hereditary nature of vascular EDS.

## Background

Patients with vascular Ehlers-Danlos syndrome (EDS) are susceptible to significant vascular complications, such as aortic and visceral arterial ruptures, aneurysms, and dissection [1]. EDS is a heterogeneous group of inherited connective tissue disorders, with its major manifestations being skin fragility, skin hyperextensibility, and joint hypermobility [2]. Types I–IV of EDS, among them at least 10 types of EDS identified to date, are most commonly observed, affecting 95% of type-specific individuals [3]. Type IV, which is referred to as Sack-Barabas syndrome, was first identified in 1936 by Sack and later modified by Barabas [4]. Here, we describe a case of repeated bleeding in a 57-year-old woman and a case of sudden onset of artery dissection in her daughter, both of whom were previously diagnosed with vascular EDS and managed at our institution.

## Case presentation

A 57-year-old woman was admitted to our emergency department due to sudden onset of left low back pain and left lower limb paralysis. Her past history included vascular EDS. Her daughter and son were both diagnosed with vascular EDS when they underwent genetic screening. On examination, she was in hypovolemic shock and had cold extremities and impaired consciousness (Glasgow coma scale 14). An urgent abdominal computed tomography (CT) scan revealed a left-sided retroperitoneal hematoma and left external iliac artery dissection.

Stent graft repair was performed under general anesthesia, which was induced with propofol, fentanyl, rocuronium, and tracheal intubation. The GORE EXCLUDER leg stent graft (16 × 70 mm; W. L. Gore and Associates, Inc., Flagstaff, AZ) was deployed, covering the left external iliac artery. Just after stent grafting, her blood pressure decreased (arterial blood pressure, 50/30 mmHg; heart rate, 91 beats/min); digital subtraction angiography (DSA) revealed a rupture of the left external iliac artery distal to the stent graft. Thus, the GORE EXCLUDER leg stent graft (12 × 100 mm) was deployed to cover the site of rupture. However, she developed hypovolemic shock (arterial blood pressure, 39/27 mmHg; heart rate, 80 beats/min) soon after the second stenting attempt, and DSA revealed rupture of the left external iliac artery distal to the second stent graft. Therefore, the GORE EXCLUDER leg stent graft (12 × 100 mm) was deployed from the end of the second stent graft to the puncture site. A completion angiogram revealed the arrest of hemorrhage from the left external iliac artery and run-off to the left popliteal artery.

Five hours postoperatively, abdominal distention increased progressively, leading to hypovolemic shock. Soon after the decision was made to attempt stent graft repair again, cardiac arrest occurred, and resuscitation attempts failed.

The 32-year-old daughter, a pharmacist, was notified of the death of her mother during the customary end-of-life conference. She experienced a sudden onset of left low back pain 6 h after her mother’s death and was admitted to our emergency department. Her past history included genetically diagnosed vascular EDS with left external iliac artery dissection, abdominal aortic aneurysm, and ileostomy after strangulating obstruction 4 years ago. On examination, she was not in hypovolemic shock: Glasgow coma scale was 15, heart rate 74 beats/min, arterial blood pressure 116/66 mmHg, oxygen saturation 100% (room air), and respiratory rate 24/min. She had marked tenderness in her lower back, and weak pulses were palpable in the bilateral dorsalis pedis. Her initial blood tests showed hemoglobin of 12.9 g/dl and a prothrombin time international normalized ratio of 1.03. An urgent abdominal CT scan revealed a right-sided retroperitoneal hematoma around the right external iliac artery and left external iliac artery dissection. Upon admission to the ICU, the only sickroom available for her was the one in which her mother had died several hours ago. As we found this inauspicious and were afraid of making her uncomfortable, we exchanged the sickroom with that of another patient. She underwent conservative therapy consisting of bed rest and antihypertensive therapy with nicardipine in the ICU. She developed no further vascular complications requiring surgical intervention, remained stable in the ICU, maintained palpable bilateral dorsalis pedis pulses throughout her hospital stay, and was discharged on the 21st hospital day. No evidence of re-bleeding was noted after discharge.

## Discussion

The prevalence of EDS type IV has been estimated to be 1:10^5^−1:10^6^ [4]. It is characterized by the deficiency of type III collagen, which is the major component of the walls of hollow structures such as the blood vessels, intestines, lungs, uterus, and skin. A clinical diagnosis of vascular EDS rests on the finding of at least two of four diagnostic criteria, including thin, translucent skin; arterial, intestinal, or uterine rupture; easy bruising; and a characteristic facial appearance [5]. Patients frequently die in their 30s and 40s, and survival beyond 50 years is exceptional [2]. In the present case, the mother had a comparatively long life (57 years) among patients with vascular EDS.

When dealing with vascular complications in patients with vascular EDS, surgical treatment is generally reserved for those presenting with imminent or frank life-threatening bleeding [6]. In fact, there appears to be a consensus of opinion for non-operative management whenever possible [7, 8]. On the other hand, there are documented reports of successful endovascular interventions, including stent delivery in vascular EDS patients with aortic aneurysms, iliac aneurysms, and hepatic artery aneurysms [5]. Perioperative management for EDS patients includes strict intraoperative control of blood pressure to avoid rupture and arterial dissection under general anesthesia, although spontaneous pneumothorax due to mechanical ventilation may occur [9, 10]. Thus, airway pressure management is important for intubated patients. All patients, even after minor surgery, should be monitored for at least 24 h postoperatively.

Bleeding in the 32-year-old daughter could have been somewhat affected by her mother’s death, possibly due to psychological stress. The cardiac impact of psychological stress historically and socially understood as boundary experiences of human life has long since become an icon [11]. For example, the etiology of Takotsubo cardiomyopathy, also known as stress-induced cardiomyopathy or broken heart syndrome, potentially involves a hyperadrenergic state associated with emotional stress [12]. Similarly, psychological stress could have been a contributing factor in the daughter. Given that vascular EDS is a genetic syndrome, the importance of psychological care for the bereaved family should not be taken lightly.

Highlighting the importance of communication skill improvements among healthcare providers, McDonagh reported that allowing family members more opportunities to speak during conferences may improve family satisfaction [13]. A previous study provides some insight into the care of bereaved family members, as discussed below [14]. Family members of 126 patients dying in 22 ICUs in France were randomly assigned to the intervention format or customary end-of-life conference. The end-of-life conference used in the intervention group had five objectives for caregivers, summarized by the mnemonic “VALUE”: “to value and appreciate what the family members said, to acknowledge the family members’ emotions, to listen, to ask questions that would allow the caregiver to understand who the patient was as a person, and to elicit questions from the family members.” The intervention resulted in reduced post-traumatic stress disorder-related symptoms and symptoms of anxiety and depression 3 months after the patient’s death. In the present case, the daughter was a pharmacist and may have understood the epidemiology and prognosis of this disorder better than non-medical individuals. Thus, interventions such as “VALUE” could have facilitated closer communication with the patient.

## Conclusions

Vascular rupture can be fatal in patients with vascular EDS. Our report underscores the need for strategic management of vascular complications to prevent rupture, as well as the importance of psychological care for the bereaved family, given the hereditary nature of vascular EDS.

## Abbreviations

CT: Computed tomography
DSA: Digital subtraction angiography
EDS: Ehlers-Danlos syndrome
ICU: Intensive care unit
